# Supplementary material for: Complexome profiling of the Chlamydomonas psb28 mutant reveals TEF5 as an early PSII assembly factor
Source: Plant Cell. 2025 Mar 17;37(6):koaf055. doi: 10.1093/plcell/koaf055 (PMC12164590; doi:10.1093/plcell/koaf055)
Supplement: koaf055_Supplementary_Data [file koaf055_supplementary_data.zip › psb28_interactive.zip!/plots/intensity/qualityPlots/peptides/_Cre05.g244950.t1.2.html]

### Cre05.g244950.t1.2

Trivial name:   
SeqCoverage [median]: 9.30  
Mass: 24.63 kDa  
  
 

| Sequence | MissedCleavage | Mass [Da] | wtIntensity | muIntensity | Score |
| --- | --- | --- | --- | --- | --- |
| AYKELTKDD | 2 | 1081.53 | 323.75 | 544.89 | 78.94 |
| EDIVVQGK | 0 | 886.48 | 0.00 | 715.49 | 68.92 |
| FTKEDIVVQGK | 1 | 1262.69 | 0.00 | 330.44 | 48.19 |
| KFTKEDIVVQGK | 2 | 1390.78 | 0.00 | 1980.55 | 69.86 |
|  |  |  | 323.7 | 3571.4 |  |
